# Oxaloacetate acid ameliorates paraquat-induced acute lung injury by alleviating oxidative stress and mitochondrial dysfunction

**DOI:** 10.3389/fphar.2022.1029775

**Published:** 2022-10-13

**Authors:** Wenwen Li, Mengxuan Li, Kaiyuan Chen, Yahui Tang, Ran Yin, Linhua Lan, Guangliang Hong

**Affiliations:** ^1^ First Clinical Medicine Institute, Wenzhou Medical University, Wenzhou, China; ^2^ Emergency Department, The First Affiliated Hospital of Wenzhou Medical University, Wenzhou, China; ^3^ Wenzhou Key Laboratory of Emergency and Disaster Medicine, Wenzhou, China

**Keywords:** oxaloacetate acid, paraquat, acute lung injury, oxidative stress, mitochondrial dysfunction, mitochondrial biogenesis

## Abstract

Acute lung injury (ALI) is the primary cause of death among patients with acute paraquat (PQ) poisoning, whereby peroxidative damage is an important mechanism underlying PQ-induced lung injury. There is a lack of effective interventional drugs for patients with PQ poisoning. Oxaloacetic acid (OAA) participates in multiple *in vivo* metabolic processes, whereby it facilitates the clearance of reactive oxygen species (ROS) and improves mitochondrial function. The study aimed to assess the protective effects of OAA on PQ-induced ALI and elucidate the underlying molecular mechanism. Our data demonstrated that OAA treatment significantly alleviated PQ-induced ALI and improved the survival rate of PQ-poisoned mice, and also alleviated PQ-induced cellular oxidative stress and mitochondrial dysfunction. OAA-mediated alleviation of PQ-induced mitochondrial dysfunction depends on the following mechanisms which may explain the above findings: 1) OAA effectively cleared intracellular ROS, inhibited ROS accumulation, and mitochondrial depolarization; 2) OAA inhibited the downregulation of L-OPA1 and MFN2 caused by PQ and promoted a dynamic balance of mitochondrial fusion and fission, and 3) the expression of PGC-1α, TFAM, COX2, and COX4I1, increased significantly following OAA intervention which improved mitochondrial respiratory functions and promoted its biogenesis and energy metabolism in damaged cells. In conclusion, OAA effectively cleared ROS and improved mitochondrial dysfunction, thereby significantly improving ALI caused by PQ poisoning and the animal survival rate. Therefore, OAA may be a potential drug for the treatment of PQ poisoning.

## 1 Introduction

Paraquat (PQ), a bipyridine heterocyclic compound, is a water-soluble herbicide (Supplementary Material A1). It is widely used in agriculture worldwide owing to its high weed control efficiency, limited environmental pollution, and low price (Gil et al., 2014). However, PQ is extremely toxic to humans and animals. PQ poisoning progresses rapidly and is associated with a high mortality rate (Zyoud, 2018). PQ poisoning causes injuries to multiple organs of the body, with the lungs being the main target. PQ actively accumulates in the alveolar epithelial cells through the polyamine uptake system, resulting in the concentration of PQ in lung tissues, which is 6–10 times higher than that in the plasma (Dinis-Oliveira et al., 2008; Dong et al., 2016). Consequently, PQ poisoning can lead to acute lung injury (ALI) and irreversible pulmonary fibrosis in the early and later stages, respectively, ultimately resulting in respiratory failure which is the major cause of death among these patients (Weng et al., 2013).

The mechanism underlying ALI due to PQ poisoning remains largely unclear; however, peroxidative damage is considered to be the key initiating factor for PQ-induced lung injury (Blanco-Ayala et al., 2014). In the cell, PQ^2+^ transfers electrons to O_2_ *via* repeated cycles of acquisition, resulting in the formation of O_2_^−^ and overproduction of reactive oxygen species (ROS). It induces cellular peroxidative damage and promotes inflammatory responses and apoptosis. Mitochondria are the most important source of intracellular ROS, and mitochondrial dysfunction plays a significant role in the pathogenesis of PQ poisoning (Liao et al., 2017). Effective interventional drugs for alleviating PQ-induced mitochondrial dysfunction and peroxidative damage are lacking.

Oxaloacetic acid (OAA), a dihydroxy acid (Supplementary Material SA2), is an important metabolic intermediate that participates in several metabolic and energy production pathways, including the citric acid cycle, gluconeogenesis, and glycolic acid cycle. OAA reportedly supports glycolysis and respiratory flux and promotes biogenesis in neuronal cells (Wilkins et al., 2016) and mitochondrial biogenesis in the mouse brain (Wilkins et al., 2014). OAA also alleviates oxidative stress. OAA prevents oxidative damage by clearing ROS in hepatocytes and maintaining the normal mitochondrial structure (Kuang et al., 2018). It also prevents alterations in cellular metabolism by inhibiting hydrogen peroxide (Long and Halliwell, 2012). Thus, OAA contributes to improving mitochondrial function, promoting biogenesis and energy metabolism, and alleviating peroxidative damage, thus implicating it as a potential interventional drug for the treatment of PQ poisoning.

In this study, we used C57BL/6J mice and human normal lung epithelial line BEAS-2B cells (B2B) to establish a PQ-induced lung injury model for investigating the role and mechanism of OAA in PQ-induced ALI.

## 2 Materials and methods

### 2.1 Animals

Male C57BL/6J mice (weight 20–25 g, 7–8 weeks) were provided by the Animal Experimental Center of Wenzhou Medical University (Wenzhou, China). Mice were housed in SPF with a 12-h light-dark cycle and were allowed free access to food and water. All animal experimental procedures were approved by the Laboratory Animal Ethics Committee of Wenzhou Medical University and Laboratory Animal Centre of Wenzhou Medical University (Wenzhou, China).

Mice were randomly divided into four groups with six in each group: 1) control group, mice received saline solution; 2) PQ group, mice received PQ (50 mg/kg); 3) protection group, OAA (10 mM, 10 mg/kg) was intraperitoneal injection into mice for three consecutive days, and PQ (50 mg/kg) was injected 1 h after the last day of OAA administration; 4) OAA group, mice received OAA (10mM, 10 mg/kg) for three consecutive days. The model was produced by intraperitoneal injection. The mice were killed at 48 h after PQ injection, and their serum, lung tissues, alveolar lavage fluid were collected for further analyses.

For survival analysis, four groups were included with 10 mice in each group as follows: 1) control group, mice received saline solution; 2) PQ group, mice received PQ (70 mg/kg); 3) protection group, a single dose of OAA (10 mM, 10 mg/kg) administered intraperitoneally for three consecutive days, and the last day administered 1 h before PQ (70 mg/kg) injection; 4) OAA group, mice received OAA (10 mM, 10 mg/kg) for three consecutive days. The death of mice in four groups was monitored within 24 h after PQ injection.

### 2.2 Wet/dry weight ratio

The right middle lobe lung tissues was excised and the wet weight was determined. Then the lung tissues were heated at 60°C for 48 h and the dry weight was determined. The lung wet/dry weight ratio was calculated to reflect the edema of lung tissue.

### 2.3 Histopathological evaluation staining

The left lung tissues were collected and fixed with 4% formaldehyde for 48 h. The tissues were embedded in paraffin wax and cut into 5 μm thick sections, and then stained with hematoxylin and eosin according to the manufacturer’s instructions (Solarbio, Beijing, China). The lung injury score was calculated according to the previously reported methods (Liu et al., 2013; Shen et al., 2017).

### 2.4 Determination of SOD, MDA, MPO, and inflammatory cytokines

The activity of SOD, the content of MDA and the activity of MPO in lung was calculated according to the manufacturer’s instructions (Nanjing Jiancheng, Nanjing, China). The content of IL-6 in BALF was determined by ELISA kits (Multi Sciences, Hangzhou, China).

### 2.5 TUNEL assay

The apoptosis of lung tissues *in situ* was determined according to the instructions of TUNEL assay kit (Roche, Indianapolis, IN, United States). The cell nuclei were stained with DAPI (Beyotim Biotechnology), and then the fluorescence microscope was used for visualization.

### 2.6 B2B cell culture and treatment

Human normal lung epithelial line BEAS-2B cells (B2B) were purchased from American Type Culture Collection, and cultured in DMEM (Gibco, Grand Island, NY, United States) containing 10% fetal bovine serum (Gibco) with 5% CO_2_ at 37°C. B2B cells were treated with different concentration of PQ (0, 200, 400, 800 and 1,600 μM) for 48 h. The results showed that cell viability was decreased in a dose-dependent manner and the dose of 400 μM was selected for subsequent experiments (Supplementary Material SB1). Next, cells were treated with different concentration of OAA (0, 2.5, 5, 10, 20 mM) and stimulated with 400 μM PQ for 48 h. We found that OAA was optimal in inhibiting PQ toxicity at a concentration of 5 mM (Supplementary Material SB2). Therefore, the concentration of 5 mM OAA was selected.

The cells were divided into the following five groups: 1) control group, the B2B cells were treated with culture medium for 48 h; 2) PQ group, the B2B cells were stimulated with PQ (400 μM) alone for 48 h; 3) protection group, the B2B cells were incubated with OAA (5 mM) and PQ (400 μM) for 48 h; 4) OAA group, the B2B cells were treated with OAA (5 mM) alone for 48 h.

### 2.7 Cell viability

The Cell Counting Kit-8 (CCK-8) assay (HY-K0301, MedChemExpress) was used to detect cell viability. 10 μL CCK-8 was added to each well, then cultured in incubator for 2 h and the absorbance was measured by a microplate reader at 450 nm.

### 2.8 Cell apoptosis

Cell apoptosis was determined by an Annexin V-FITC/PI apoptosis detection kit (Beyotim Biotechnology). Briefly, the cells were resuspended in 195 μL of binding buffer, and stained with 5 μL Annexin V-FITC and 10 μL propidium iodide (PI), then incubated at room temperature for 15 min in the dark. The cell apoptosis was determined by flow cytometry.

### 2.9 Measurement of intracellular reactive oxygen species levels

The intracellular ROS levels were determined according to the instructions of the Reactive Oxygen Species Assay Kit (Beyotim Biotechnology). Briefly, cells were incubated with DCFH-DA for 20 min in the dark at 37°C and then analyzed by flow cytometry.

### 2.10 Measurement of mitochondrial membrane potential

Mitochondrial membrane potential (MMP) was studied with the JC-1 probe, and determined by mitochondrial membrane potential assay kit (Beyotim Biotechnology). Briefly, cells were incubated with an equal volume of a JC-1 working solution for 20 min in the dark at 37°C and measured by flow cytometry. The ratio of JC-1 monomer to aggregate indirectly reflected the MMP.

### 2.11 MitoTracker red staining

The changes in mitochondrial dynamics were detected by using MitoTracker Red CMXRos in B2B cells. After treatment with PQ or OAA for 48 h, cells were incubated with 100 nM MitoTracker Red (#9082, Cell Signaling Technology) for 30 min at 37°C. Then, cells were fixed with 4% paraformaldehyde for 10 min at −20°C and the nuclei were stained with DAPI (Beyotim Biotechnology), Finally, subsequent images were obtained by fluorescence microscope.

### 2.12 Cell metabolic ability assay

The oxygen consumption rate (OCR) was measured according to the instructions of Seahorse extracellular flux analyzer (Seahorse Bioscience, Billerica, MA, United States). Briefly, B2B cells were seeded into 96-well Seahorse XF plates, and sequentially injected oligomycin, carbonyl cyanide-4 (trifluoromethoxy) phenylhydrazone (FCCP) and Rotenone and Antimycin according to manufacturer’s instructions. Then, mitochondrial respiration parameters were calculated based on oxygen consumption.

### 2.13 Real-time PCR analysis

The total RNA of B2B cells was extracted according to the instructions of the RNA kits (Tiangen Biotech, Beijing, China). Then the RNA was used to synthesize the cDNA by reverse transcription kit (Thermo Scientific, United States). Subsequently, SYBR Premix Ex Taq II (Bio-red, United States) was used for real-time PCR. The following primers were used in our study: TFAM, F 5'-TTC​CAA​GAA​GCT​AAG​GGT​GAT​T-3' and R 5'-AGA​AGA​TCC​TTT​CGT​CCA​ACT​T-3'; PGC-1, F 5'-CAG​AGA​GTA​TGA​GAA​GCG​AGA​G-3' and R 5'-AGC​ATC​ACA​GGT​ATA​ACG​GTA​G-3'; COX4l1, F 5'-CCA​GAA​GGC​ATT​GAA​GGA​GAA​GGA​G-3' and R 5'-CCA​CAA​CCG​TCT​TCC​ACT​CGT​TC-3'; COX2, F 5'-CGC​ATC​CTT​TAC​ATA​ACA​GAC​G-3' and R 5'- TAG​GAG​TTG​AAG​ATT​AGT​CCG​C-3' (Sandon Biotech, Shanghai, China). PCR conditions were 1min at 95°C, followed by 40 cycles of 10 s at 95°C and 30 s at 60°C.

### 2.14 Western blotting analysis

The protein of lung tissue or cells were extracted by lysis buffer (Solarbio). The protein samples (30 ug) were separated by using 12% SDS-PAGE gels and then transferred to polyvinylidene fluoride membranes (Sigma, Shanghai, China). Membranes were blocked for 10 min at room temperature with 5% BSA blocking buffer (Shanghai Yamei, Shanghai, China) and incubated for 24 h at 4°C with primary antibody. Subsequent, membranes were washed with Tris-buffered saline containing Tween 20 (Solarbio, Beijing, China) at least 3 times and were incubated for 1 h at room temperature with HRP-conjugated secondary antibodies (1:5,000 dilution, Beyotim Biotechnology, Shanghai, China). After washing 3 times, immunoreactivity was visualized by Chemiluminescent Substrate (Thermo Scientific, United States). The following primary antibody were used: OPA1 antibody (1:1,000, 80471, Cell Signaling Technology, United States), MFN2 antibody (1:1,000, ab56889, abcam, England), CHOP antibody (1:1,000, 15204-1-AP, proteintech, China), TFAM antibody (1:1,000, 8,076, Cell Signaling Technology), Bcl-2 antibody (1:2000, ab182858, abcam), Bax antibody (1:1,000, ab32503, abcam), Cleaved-caspase-3 antibody (1:500, ab32042, abcam), Caspase3 antibody (1:1,000, 14220, Cell Signaling Technology) and β-action (1:5,000, 20536-1-AP, proteintech).

### 2.15 Statistical analysis

Experimental data were performed by GraphPad Prism 9 software and presented as mean ± standard deviation (SD). All experiments were performed at least in triplicate tests. All statistical analyses were performed using *t*-test to compare the averages between the two groups. The level of statistically significant was set at *p* < 0.05.

## 3 Results

### 3.1 Oxaloacetic acid prolongs mice survival following paraquat poisoning

**FIGURE 1:**
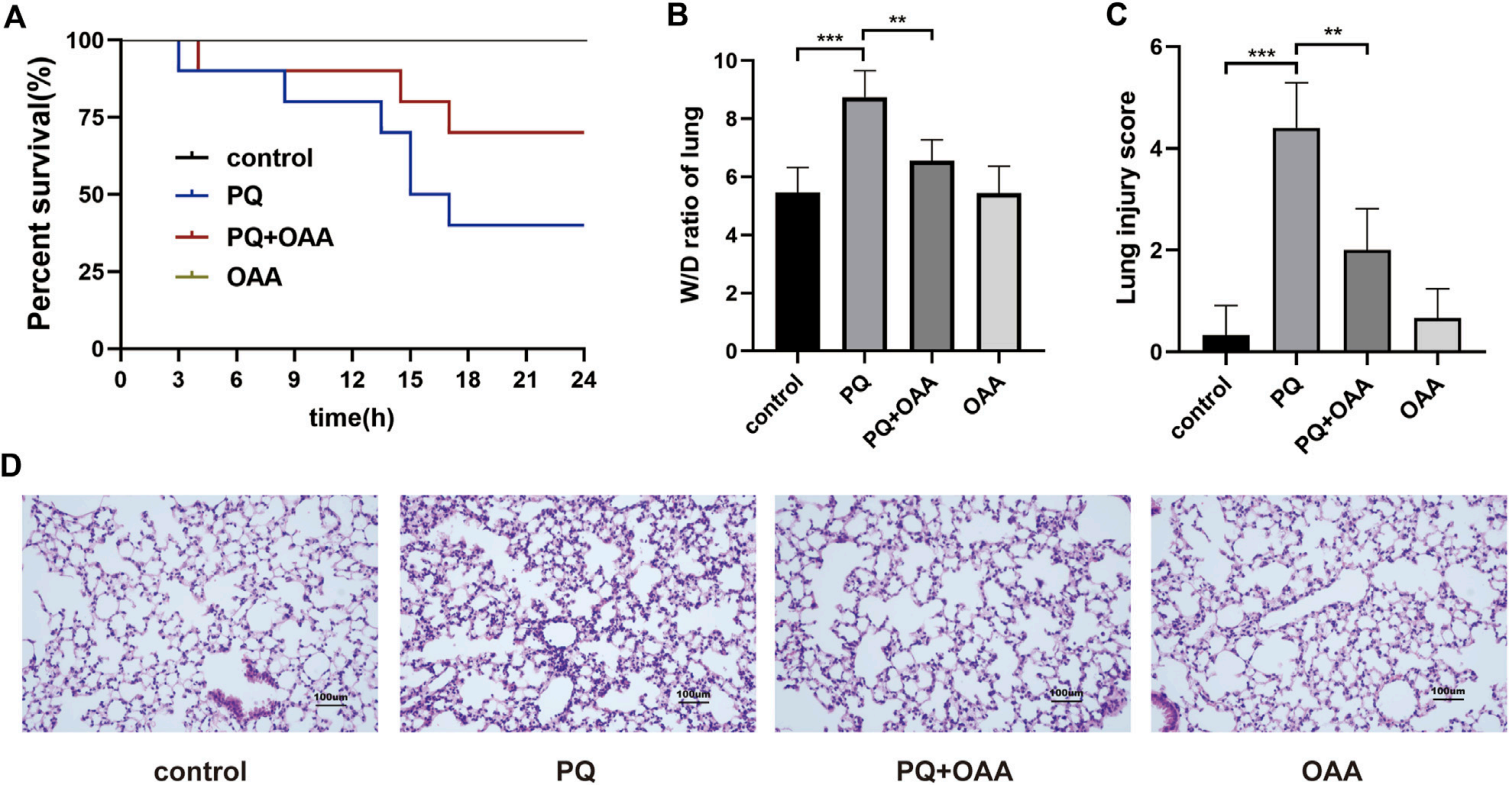
Protective effect of OAA in mice after PQ poisoning. **(A)** Kaplan-Meier survival curve. **(B)** The lung W/D ratio. **(C)** Lung injury score. **(D)** HE staining of mice lung tissue ( × 200). Data are presented as mean ± SD (*n* = 6). *p＜0.05, **p＜0.01, ***p＜0.001, ****p＜0.0001. PQ, paraquat; OAA, oxaloacetate acid.

To investigate the protective effects of OAA, we examined the survival rate and the status of mice after PQ poisoning and OAA treatment. As shown in Figure 1A, 24 h post-PQ and OAA treatment, the survival rate of the protection group mice was nearly 66.7%, which was significantly higher than that in the PQ group (33.3%, *p* < 0.05). The state of mice in the PQ group was worse than that in the protection group, evidenced by the loss of hair luster, a distinct reduction in activity, and an increased respiratory rate. These results suggested that OAA significantly improved survival and alleviated the state of mice following PQ poisoning.

### 3.2 Oxaloacetic acid reduces the wet/dry weight ratio of mice lungs after paraquat poisoning

The wet/dry (W/D) weight ratio of mice lungs is considered an important indicator for evaluating lung edema. As shown in Figure 1B, the 48 h of PQ treatment caused a significant increase in the W/D ratio as compared to the control and OAA groups (*p* < 0.05). However, OAA intervention resulted in a significant decrease in the W/D ratio of lungs in mice after PQ poisoning (*p* < 0.05). These results suggested that OAA treatment alleviated PQ-induced lung edema.

### 3.3 Oxaloacetic acid alleviates paraquat-induced histopathological damage in lungs tissues of mice

To examine the severity of lung injury, HE staining was performed on day 2 after PQ poisoning. As shown in Figure 1D, the structures were normal without signs of inflammation in the control and OAA groups. In contrast, the application of PQ alone caused a markedly high inflammatory cell infiltration and obvious interstitial inflammation. The administration of OAA significantly alleviated PQ-induced inflammatory reactions. The pathological score of lung injury in the PQ group was significantly higher than those in the control and OAA groups (*p* < 0.05), whereas, the protection group showed a relatively lower pathological score (Figure 1C, *p* < 0.05). These results suggested that OAA treatment reduced PQ-induced histopathological damage to lung tissues.

### 3.4 Oxaloacetic acid inhibits paraquat-induced oxidative stress and inflammation factors in mice

**FIGURE 2:**
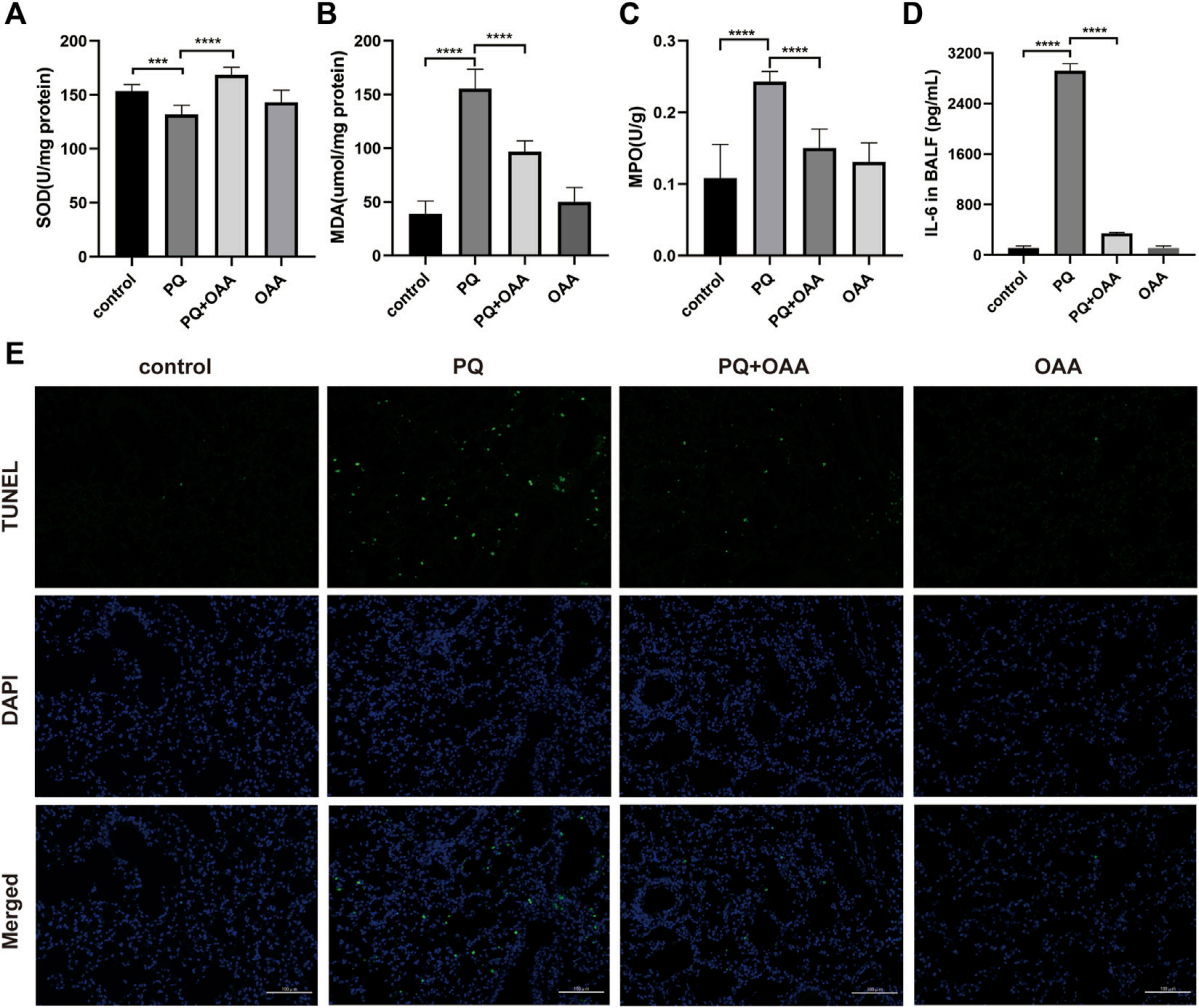
OAA alleviates oxidative stress, inflammation factor and apoptosis in mice after PQ poisoning. **(A)** SOD activity in mice lungs. **(B)** MDA levels in mice lungs. **(C)** MPO activity in mice lungs. **(D)** IL-6 levels in BALF. **(E)** Representative images of TUNEL staining (green) and DAPI staining (blue) ( × 200). Data are presented as mean ± SD (*n* = 6). *p＜0.05, **p＜0.01, ***p＜0.001, ****p＜0.0001. PQ, paraquat; OAA, oxaloacetate acid; BALF, bronchoalveolar lavage fluid.

To investigate the effects of OAA on oxidative stress and inflammation factors after PQ poisoning, the contents of SOD, MDA, and MPO were assessed in the mice lungs and that of IL-6 in the bronchoalveolar lavage fluid (BALF). As shown in Figures 2A,B, the content of SOD with anti-oxidative bioactivity decreased significantly in mice lungs in the PQ group as compared to the control and OAA groups (*p* < 0.05), whereas MDA levels with lipid peroxidation bioactivity increased significantly (*p* < 0.05). After OAA treatment, the levels of SOD increased significantly while those of MDA decreased significantly as compared to the PQ group (*p* < 0.05). As shown in Figures 2C,D, compared to the PQ group, the levels of MPO in the lungs and those of IL-6 in BALF increased significantly in the control and OAA groups (*p* < 0.05) but decreased significantly in the protection group (*p* < 0.05). These results indicated that OAA treatment inhibited PQ-induced oxidative stress and inflammation *in vivo*.

### 3.5 Oxaloacetic acid inhibits paraquat-induced apoptosis in lungs tissues of mice

We examined apoptosis and DNA damage in lung tissues by TUNEL staining assay. Figure 2E shows that PQ exposure dramatically elevated apoptosis and DNA damage relative to the control and OAA groups (*p* < 0.05). However, OAA treatment after PQ poisoning significantly reduced the number of TUNEL-positive lung cells as compared to those in the PQ group (*p* < 0.05). The above-mentioned results indicated that OAA could inhibit PQ-induced apoptosis.

### 3.6 Protective effects of oxaloacetic acid on paraquat-induced damage in B2B cells

**FIGURE 3:**
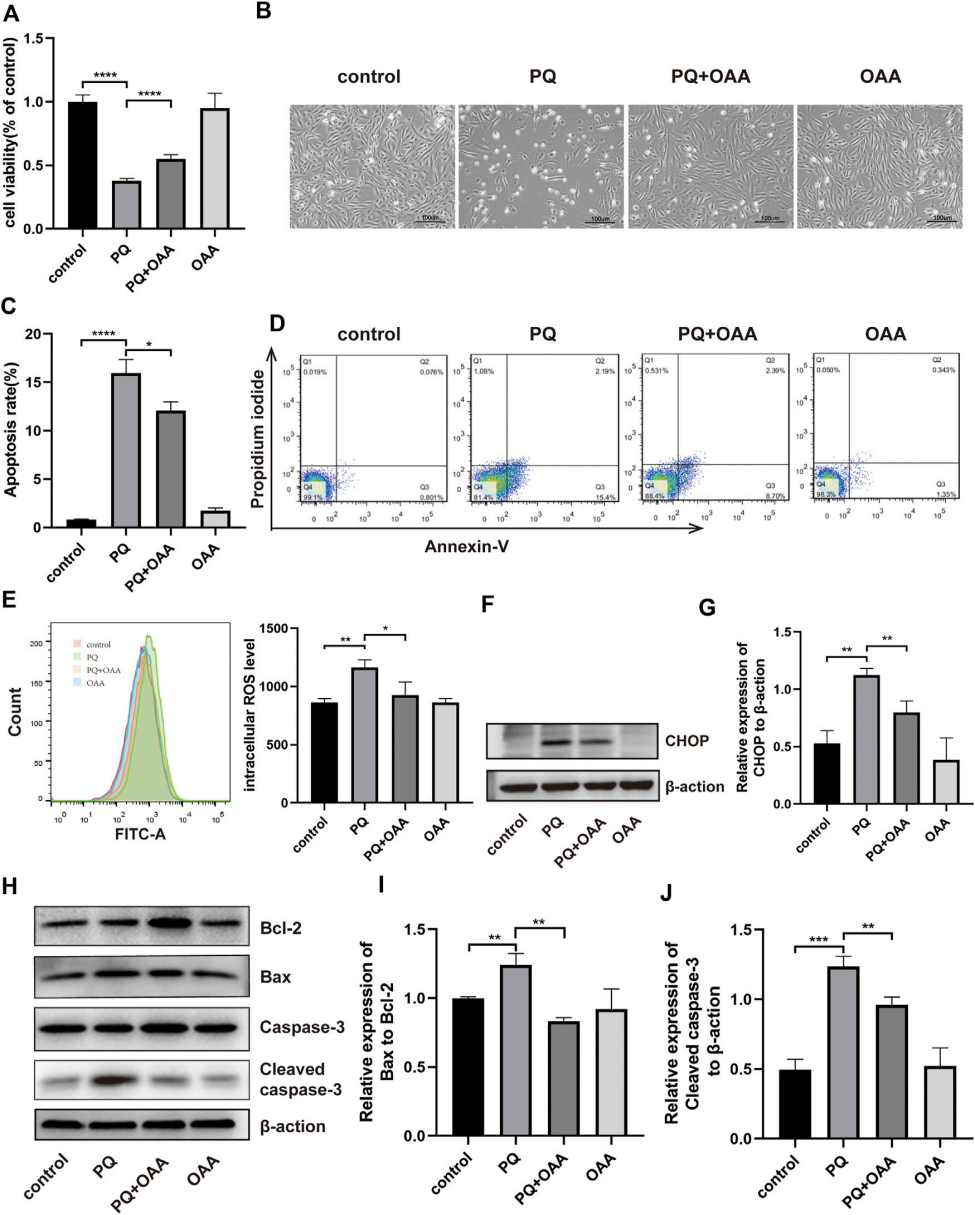
OAA alleviates PQ-induced cytotoxicity, apoptosis and oxidative stress in B2B cells. **(A)** The cell viability was evaluated by CCK-8 assay. **(B)** Morphological changes of B2B cells under the optical microscope. **(C,D)** Cell apoptosis was detected by flow cytometry. **(E)** Intracellular ROS levels was measured by flow cytometry. **(F,G)** The expression levels of CHOP protein was detected by WB assay. **(H–J)** The expression levels of Bcl2, Bax, Caspase-3 and Cleaved Caspase-3 protein was detected by WB assay. Data are presented as mean ± SD (*n* = 3). *p＜0.05, **p＜0.01, ***p＜0.001, ****p＜0.0001. PQ, paraquat; OAA, oxaloacetate acid.

To further confirm the protective effects of OAA in B2B cells, the cell viability was assessed using the CCK-8 kit and Annexin V/PI assay. As shown in Figure 3A, after PQ exposure, the viability of cells decreased significantly as compared to those in the control and OAA groups (*p* < 0.05). However, it increased significantly after co-treatment with OAA and PQ as compared to stimulation with PQ alone (*p* < 0.05). The cellular state was examined under an optical microscope. As shown in Figure 3B, the number of B2B cells reduced following PQ treatment, and their morphology was altered; however, this change was attenuated after OAA intervention. As shown in Figures 3C,D, the Annexin V/PI assay revealed that the number of apoptotic cells in the PQ group was higher than those in the control and OAA groups (*p* < 0.05) and decreased significantly following OAA treatment (*p* < 0.05).

### 3.7 Oxaloacetic acid reduces reactive oxygen species generation and alleviates mitochondrial-dependent apoptosis induced by paraquat in B2B cells

Oxidative stress may be the main cause of PQ-induced ALI; thus, we examined intracellular ROS levels in B2B cells. As shown in Figure 3E, intracellular ROS levels were significantly high in the PQ group relative to the control and OAA groups (*p* < 0.05); these levels were markedly lower in the protection group as compared to the PQ group (*p* < 0.05).

ROS overproduction can lead to mitochondrial-dependent apoptosis (Bock and Tait, 2020); therefore, we further examined apoptosis by WB assay (Figures 3H–J). These results demonstrated that the ratio of protein expression of proapoptotic–Bax and anti-apoptotic–Bcl-2 decreased significantly in the protection group as compared to the PQ group (*p* < 0.05). The expression of proapoptotic protein–cleaved Caspase-3 (CC3) also decreased significantly in the protection group (*p* < 0.05). CHOP has been previously reported as a crucial upstream transcription factor regulating the expression of the Bcl-2 family of proteins (Hu et al., 2018). Thus we examined CHOP expression in B2B cells by WB assay (Figures 3F,G). PQ treatment significantly promoted the protein expression of CHOP (*p* < 0.05), which decreased following OAA treatment (*p* < 0.05). These results suggested that PQ enhanced ROS generation and activated the mitochondrial-dependent apoptotic pathway; however, these effects were inhibited following OAA treatment.

### 3.8 Oxaloacetic acid alleviates paraquat-induced mitochondrial depolarization in B2B cells

**FIGURE 4:**
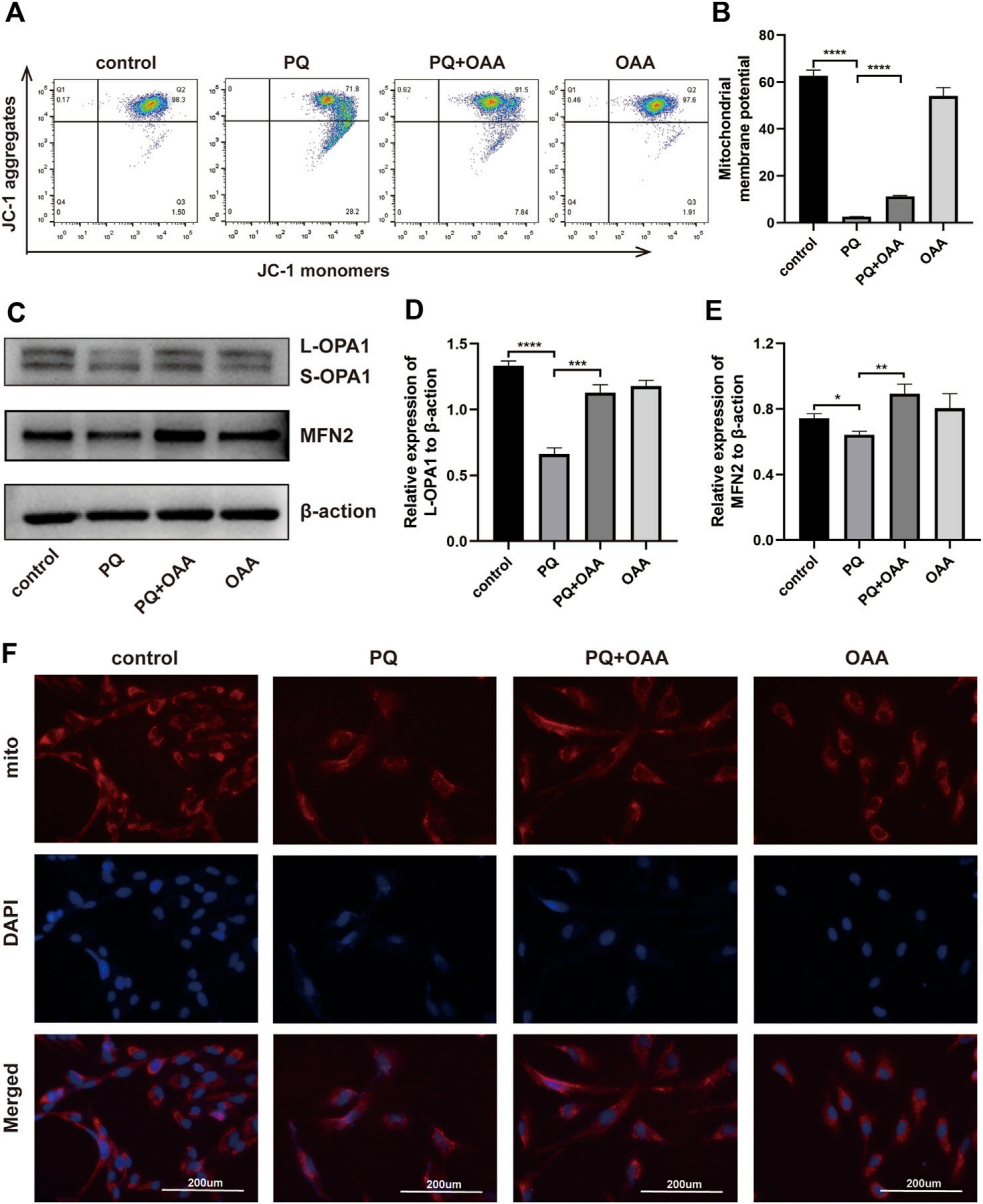
OAA improves PQ-induced mitochondrial function in B2B cells. **(A,B)** After JC-1 staining, MMP was determined by flow cytometry. **(C–E)** The expression levels of L-OPA1, S-OPA1 and MFN2 protein was detected by WB assay. **(F)** After MitoTracker staining, the changes in mitochondrial dynamics were detected by immunofluorescence. Mitochondrial stained with MitoTracker (red) and nuclei were stained with DAPI (blue). Data are presented as mean ± SD (*n* = 3). *p＜0.05, **p＜0.01, ***p＜0.001, ****p＜0.0001. PQ, paraquat; OAA, oxaloacetate acid; mito, mitochondrial.

Mitochondrial membrane potential (MMP) reflects the functional status of mitochondria and is linked to ATP production. To investigate the mechanisms underlying the protective effects of OAA, we measured the MMP by JC-1. JC-1 aggregation implies a high MMP, whereas its presence as monomers indicates a low MMP. As shown in Figures 4 A,B, relative to the control and OAA groups, MMP decreased significantly when the cells were exposed to PQ alone (*p* < 0.05). However, it increased significantly following OAA treatment (*p* < 0.05). These results suggested that OAA alleviated PQ-induced MMP decline and mitochondrial depolarization.

### 3.9 Oxaloacetic acid promotes mitochondrial dynamics in B2B cells

Mitochondrial dynamics maintain their physiological functions (Mishra and Chan, 2014; Persad and Lopaschuk, 2022), and abnormalities herein may lead to cell death due to mitochondrial damage. To further examine whether OAA could promote mitochondrial fusion to maintain mitochondrial dynamics, we performed WB and MitoTracker Red assay. As shown in Figures 4C–E, the expressions of mitochondrial fusion-related protein, long OPA1 (L-OPA1), and fusion-promoting protein, MFN2, decreased significantly in the PQ group as compared to the control and OAA groups (*p* < 0.05). After treatment with OAA, the levels of L-OPA1 and MFN2 expression increased significantly (*p* < 0.05). The results of the MitoTracker assay revealed that after PQ exposure, the normal mitochondrial morphology was lost and these were fragmented. OAA treatment alleviated the above effects of PQ (Figure 4F). These results suggested that PQ caused an imbalance between mitochondrial fission and fusion, and OAA effectively promoted normal mitochondrial fission and fusion.

### 3.10 Oxaloacetic acid promotes mitochondrial biogenesis and alleviates mitochondrial respiratory dysfunction induced by paraquat in B2B cells

**FIGURE 5:**
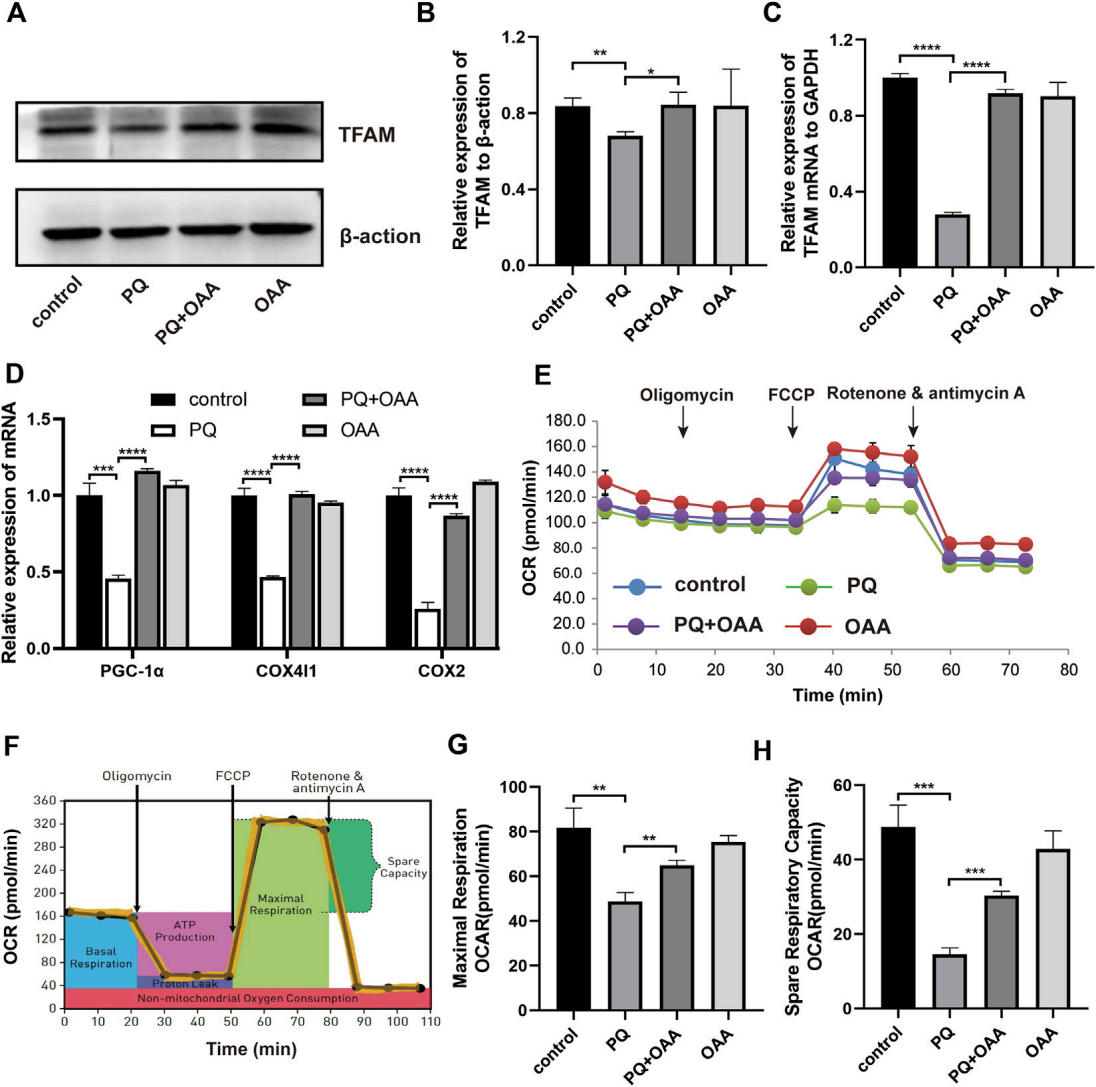
OAA promotes mitochondrial biogenesis and alleviates mitochondrial respiration dysfunction caused by PQ in B2B cells. **(A,B)** The expression levels of TFAM protein was detected by WB assay. **(C,D)** The mRNA expression levels of TFAM, PGC-1α, COX4l1, and COX2 was detected by real-time PCR assay. **(E,F)** OCR was detected by mitochondrial stress assay. **(G,H)** Maximal respiration and spare respiration capacity were calculated after oligomycin and FCCP treatment. Data are presented as mean ± SD (*n* = 3). *p＜0.05, **p＜0.01, ***p＜0.001, ****p＜0.0001. PQ, paraquat; OAA, oxaloacetate acid; OCR: oxygen consumption rate; FCCP, carbonyl cyanide-4 (trifluoromethoxy) phenylhydrazone.

To further elucidate the mechanism underlying OAA-mediated alleviation of PQ-induced ALI, we assessed mitochondrial biogenesis and respiration functions by WB, real-time PCR, and mitochondrial stress assay. As shown in Figures A–C, the levels of protein and mRNA expression of the mitochondrial biogenesis biomarker, TFAM, were elevated significantly in the protection group as compared to the PQ group (*p* < 0.05). Similarly, mitochondrial biogenesis-related mRNA expression of PGC-1α, COX4I1, and COX2 increased significantly in the protection group as compared to the PQ group (Figure 5D, *p* < 0.05). Moreover, the mitochondrial oxygen consumption rate (OCR) was assessed (Figures 5E,F). The results demonstrated that PQ caused a significant decrease in the maximal respiration and spare respiration capacities, while OAA alleviated mitochondrial respiratory dysfunction by increasing maximal respiration and spare respiration capacities (Figures 5G,H, *p* < 0.05). These results suggested that PQ disrupted mitochondrial biogenesis and mitochondrial respiratory function, while OAA intervention alleviated the above symptoms.

## 4 Discussion

To date, an effective intervention drug for the treatment of PQ poisoning is lacking. In our study, OAA was found to effectively reduce mortality and improve lung injury following PQ poisoning in mice. This protective effect could be attributed to the fact that OAA inhibited the production of cellular ROS, promoted the maintenance of mitochondrial dynamics, and regulated mitochondrial biogenesis and energy metabolism. It effectively alleviated PQ-induced oxidative stress and mitochondrial dysfunction. This study is the first to describe the protective role and the mechanism underlying the antagonizing effects of the endogenous metabolite, OAA, in ALI following PQ poisoning. The findings provide new ideas for identifying clinical therapeutic drugs for the treatment of PQ poisoning.

The mechanisms of PQ-induced lung injury are complex and diverse, and several reports demonstrated that mitochondrial dysfunction is one of the most important mechanism of PQ toxicity (Witschi et al., 1977; Wang et al., 2018; Bora et al., 2021). The specific mechanism of mitochondrial damage by PQ exposure remains unclear, however, the following aspects have been reported: 1) oxidative stress: PQ can cause cellular oxidative stress and oxidative damage through the production of excessive ROS, consequently leading to mitochondrial dysfunction (He et al., 2016; Zhang et al., 2021). This is consistent with our findings which showed that PQ increased oxidative stress and inflammatory factors to promote apoptosis *in vivo*, while *in vitro*, PQ exposure led to ROS production and induced mitochondrial-mediated apoptosis, herein. 2) Altered MMP: ROS interacts with the mitochondrial membrane components to alter mitochondrial permeability and decrease MMP (Chen et al., 2012; Chowdhury et al., 2020), which breaks the balance of apoptosis regulating proteins (Bax/Bcl-2) and induces apoptosis (Fei and Ethell, 2008; Zhang et al., 2019). It is reported that PQ decreased MMP in rat lungs and mitochondrial-mediated apoptosis in A549 cells (Cui et al., 2019; Zhang et al., 2020; Zhang et al., 2021). Our findings revealed that PQ caused an increase in ROS production, enhanced the Bax/Bcl-2 ratio and altered MMP. Meanwhile, PQ also caused an increase in the levels of CHOP, an upstream transcription factor of the Bcl-2 protein family, thus exacerbating the Bax/Bcl-2 ratio, which in turn reduced the MMP. 3) Disruption of mitochondrial dynamics: mitochondrial dynamics include mitochondrial fusion and fission, whereby the former is mediated by optic atrophy 1 (OPA1) and mitofusin 2 (MFN2). Studies have shown that ROS-mediated mitochondrial fission lead to PQ-induced neuronal cell injury (Chen et al., 2021). Promoting MFN2-mediated mitochondrial fusion protects against PQ-induced lung epithelial cell injury (Liu et al., 2022). Our findings confirmed that PQ downregulated the protein expression of long-chain OPA1 and MFN2, thereby inhibiting mitochondrial fusion. 4) Inhibited mitochondrial biogenesis and energy metabolism: The process of mitochondrial biogenesis is complex and involves multiple mitochondrial and nuclear proteins (Zhu et al., 2013). The peroxisome value-added substance-activated receptor-γ coactivator (PGC-1α) initiates mitochondrial biogenesis (Rius-Perez et al., 2020) and further stimulate mitochondrial transcription factor A (TFAM), responsible for mitochondrial DNA transcription, replication, and stabilization (Gleyzer et al., 2005; Scarpulla et al., 2012). And the mitochondrial electron transport chain, consisting of complexes I–IV (COXI-IV), is the basis of ATP production (Czerniczyniec et al., 2015). Our findings showed that PQ inhibited the expression of TFAM, PGC-1α, COX2, and COX4I1 and suppressed mitochondrial respiratory function, leading to mitochondrial damage.

Oxaloacetate (OAA) is an endogenous metabolic intermediate involved in several metabolic processes, as an example OAA is produced from malic acid catalyzed by malate dehydrogenase, and then OAA is condensed with acetyl coenzyme A to produces citric acid, thus potentially stimulating glycolytic flux and increasing mitochondrial mass (Hsu et al., 2022). OAA is closely related to mitochondrial function. It exerts antagonistic effects on oxidative stress, promotes biogenesis and energy metabolism, and enhances mitochondrial energy flux (Wilkins et al., 2014; Jiao et al., 2017; Kuang et al., 2018; Merlen et al., 2019). However, the role and mechanism of OAA in ALI following PQ exposure remain unclear. Several studies have shown that OAA metabolism is associated with the production of NADPH (Abrego et al., 2017; Ma et al., 2022), which can act as a ROS scavenger (Ito et al., 2021), and this may be related to OAA antagonism of oxidative stress caused by PQ. In our study, OAA was found to exert protective effects on PQ-induced lung injury, possibly through inhibited oxidative stress and improved mitochondrial function. On the one hand, OAA reduced intracellular ROS levels after PQ treatment, increased the Bax/Bcl-2 ratio and Cleaved Caspase-3 protein expression, and inhibited mitochondria-mediated apoptosis, indicating that improvement in oxidative stress and inhibition of apoptosis and cell injury. On the other hand, OAA increased MMP after PQ treatment, thus inhibiting mitochondrial damage, upregulating L-OPA1 and MFN2 expression to promote mitochondrial fusion, upregulating the levels of TFAM, PGC-1α, COX2, and COX4I1, and increasing maximal and spare respiratory capacities to promote mitochondrial biogenesis and energy metabolism, indicating improved mitochondrial function and alleviation of PQ induced cellular damage.

In conclusion, OAA could clear intracellular ROS, reduce intracellular oxidative stress, maintain mitochondrial homeostasis, regulate mitochondrial biogenesis and energy metabolism, and improve mitochondrial dysfunction. OAA plays an important role in alleviating ALI due to PQ poisoning.

## 5 Conclusion

In summary, although the specific mechanism underlying PQ-induced lung injury is unclear, it is generally accepted that oxidative stress worsens it. In this study, OAA was found to exert a significant protective effect on PQ poisoning-induced ALI. This effect was attributed to the fact that OAA could effectively alleviate PQ-induced mitochondrial damage and dysfunction. First, OAA could clear ROS and attenuate oxidative stress and mitochondrial polarization. Second, OAA promoted mitochondrial fusion and its dynamic balance by upregulating L-OPA1 and MFN2 levels. Finally, OAA also improved mitochondrial biogenesis and energy metabolism by upregulating TFAM, PGC-1α, COX2, and COX4I2. Therefore, OAA is expected to be an effective drug for ROS clearance and improving mitochondrial function in PQ-induced ALI.

## Data Availability

The original contributions presented in the study are included in the article/Supplementary Material, further inquiries can be directed to the corresponding author.

## References

[B1] Abrego J. Gunda V. Vernucci E. Shukla S. K. King R. J. Dasgupta A. (2017). GOT1-mediated anaplerotic glutamine metabolism regulates chronic acidosis stress in pancreatic cancer cells. Cancer Lett. 400, 37–46. 10.1016/j.canlet.2017.04.029 28455244 PMC5488721

[B2] Blanco-Ayala T. Anderica-Romero A. C. Pedraza-Chaverri J. (2014). New insights into antioxidant strategies against paraquat toxicity. Free Radic. Res. 48, 623–640. 10.3109/10715762.2014.899694 24593876

[B3] Bock F. J. Tait S. W. G. (2020). Mitochondria as multifaceted regulators of cell death. Nat. Rev. Mol. Cell. Biol. 21, 85–100. 10.1038/s41580-019-0173-8 31636403

[B4] Bora S. Vardhan G. S. H. Deka N. Khataniar L. Gogoi D. Baruah A. (2021). Paraquat exposure over generation affects lifespan and reproduction through mitochondrial disruption in *C. elegans* . Toxicology 447, 152632. 10.1016/j.tox.2020.152632 33197508

[B5] Chen N. Guo Z. Luo Z. Zheng F. Shao W. Yu G. (2021). Drp1-mediated mitochondrial fission contributes to mitophagy in paraquat-induced neuronal cell damage. Environ. Pollut. 272, 116413. 10.1016/j.envpol.2020.116413 33422762

[B6] Chen Y. W. Yang Y. T. Hung D. Z. Su C. C. Chen K. L. (2012). Paraquat induces lung alveolar epithelial cell apoptosis via Nrf-2-regulated mitochondrial dysfunction and ER stress. Arch. Toxicol. 86, 1547–1558. 10.1007/s00204-012-0873-8 22678742

[B7] Chowdhury A. R. Zielonka J. Kalyanaraman B. Hartley R. C. Murphy M. P. Avadhani N. G. (2020). Mitochondria-targeted paraquat and metformin mediate ROS production to induce multiple pathways of retrograde signaling: A dose-dependent phenomenon. Redox Biol. 36, 101606. 10.1016/j.redox.2020.101606 32604037 PMC7327929

[B8] Cui S. Nian Q. Chen G. Wang X. Zhang J. Qiu J. (2019). Ghrelin ameliorates A549 cell apoptosis caused by paraquat via p38-MAPK regulated mitochondrial apoptotic pathway. Toxicology 426, 152267. 10.1016/j.tox.2019.152267 31381934

[B9] Czerniczyniec A. Lanza E. M. Karadayian A. G. Bustamante J. Lores-Arnaiz S. (2015). Impairment of striatal mitochondrial function by acute paraquat poisoning. J. Bioenerg. Biomembr. 47, 395–408. 10.1007/s10863-015-9624-x 26350412

[B10] Dinis-Oliveira R. J. Duarte J. A. Sanchez-Navarro A. Remiao F. Bastos M. L. Carvalho F. (2008). Paraquat poisonings: Mechanisms of lung toxicity, clinical features, and treatment. Crit. Rev. Toxicol. 38, 13–71. 10.1080/10408440701669959 18161502

[B11] Dong S. Hu H. Wang Y. Xu Z. Zha Y. Cai X. (2016). A pqr2 mutant encodes a defective polyamine transporter and is negatively affected by ABA for paraquat resistance in *Arabidopsis thaliana* . J. Plant Res. 129, 899–907. 10.1007/s10265-016-0819-y 27229891

[B12] Fei Q. Ethell D. W. (2008). Maneb potentiates paraquat neurotoxicity by inducing key Bcl-2 family members. J. Neurochem. 105, 2091–2097. 10.1111/j.1471-4159.2008.05293.x 18266926

[B13] Gil H. W. Hong J. R. Jang S. H. Hong S. Y. (2014). Diagnostic and therapeutic approach for acute paraquat intoxication. J. Korean Med. Sci. 29, 1441–1449. 10.3346/jkms.2014.29.11.1441 25408572 PMC4234908

[B14] Gleyzer N. Vercauteren K. Scarpulla R. C. (2005). Control of mitochondrial transcription specificity factors (TFB1M and TFB2M) by nuclear respiratory factors (NRF-1 and NRF-2) and PGC-1 family coactivators. Mol. Cell. Biol. 25, 1354–1366. 10.1128/MCB.25.4.1354-1366.2005 15684387 PMC548005

[B15] He Y. Zou L. Zhou Y. Hu H. Yao R. Jiang Y. (2016). Adiponectin ameliorates the apoptotic effects of paraquat on alveolar type cells via improvements in mitochondrial function. Mol. Med. Rep. 14, 746–752. 10.3892/mmr.2016.5328 27220901 PMC4918546

[B16] Hsu H. P. Chu P. Y. Chang T. M. Huang K. W. Hung W. C. Jiang S. S. (2022). Mitochondrial phosphoenolpyruvate carboxykinase promotes tumor growth in estrogen receptor-positive breast cancer via regulation of the mTOR pathway. Cancer Med. 10.1002/cam4.4969 Epub ahead of print PMC9883444 35757841

[B17] Hu H. Tian M. Ding C. Yu S. (2018). The C/EBP homologous protein (CHOP) transcription factor functions in endoplasmic reticulum stress-induced apoptosis and microbial infection. Front. Immunol. 9, 3083. 10.3389/fimmu.2018.03083 30662442 PMC6328441

[B18] Ito H. Nakamae I. Kato J. Y. Yoneda-Kato N. (2021). Stabilization of fatty acid synthesis enzyme acetyl-CoA carboxylase 1 suppresses acute myeloid leukemia development. J. Clin. Invest. 131, 141529. 10.1172/JCI141529 34128473 PMC8203453

[B19] Jiao Y. Ji L. Kuang Y. Yang Q. (2017). Cytotoxic effect of oxaloacetate on HepG2-human hepatic carcinoma cells via apoptosis and ROS accumulation. Neoplasma 64, 192–198. 10.4149/neo_2017_204 28043145

[B20] Kuang Y. Han X. Xu M. Wang Y. Zhao Y. Yang Q. (2018). Oxaloacetate ameliorates chemical liver injury via oxidative stress reduction and enhancement of bioenergetic fluxes. Int. J. Mol. Sci. 19, E1626. 10.3390/ijms19061626 29857490 PMC6032239

[B21] Liao P. H. Hsu H. H. Chen T. S. Chen M. C. Day C. H. Tu C. C. (2017). Phosphorylation of cofilin-1 by ERK confers HDAC inhibitor resistance in hepatocellular carcinoma cells via decreased ROS-mediated mitochondria injury. Oncogene 36, 1978–1990. 10.1038/onc.2016.357 27748761

[B22] Liu C. Sun Z. Wang M. Yang Z. Zhang W. Ren Y. (2022). Mitoquinone mitigates paraquat-induced A549 lung epithelial cell injury by promoting MFN1/MFN2-mediated mitochondrial fusion. J. Biochem. Mol. Toxicol. 36, e23127. 10.1002/jbt.23127 35686354

[B23] Liu Z. N. Zhao M. Zheng Q. Zhao H. Y. Hou W. J. Bai S. L. (2013). Inhibitory effects of rosiglitazone on paraquat-induced acute lung injury in rats. Acta Pharmacol. Sin. 34, 1317–1324. 10.1038/aps.2013.65 23933652 PMC4002157

[B24] Long L. H. Halliwell B. (2012). The effects of oxaloacetate on hydrogen peroxide generation from ascorbate and epigallocatechin gallate in cell culture media: Potential for altering cell metabolism. Biochem. Biophys. Res. Commun. 417, 446–450. 10.1016/j.bbrc.2011.11.136 22166196

[B25] Ma N. Shangguan F. Zhou H. Huang H. Lei J. An J. (2022). 6-methoxydihydroavicine, the alkaloid extracted from Macleaya cordata (Willd.) R. Br. (Papaveraceae), triggers RIPK1/Caspase-dependent cell death in pancreatic cancer cells through the disruption of oxaloacetic acid metabolism and accumulation of reactive oxygen species. Phytomedicine. 102, 154164. 10.1016/j.phymed.2022.154164 35597026

[B26] Merlen G. Raymond V. A. Cassim S. Lapierre P. Bilodeau M. (2019). Oxaloacetate protects rat liver from experimental warm ischemia/reperfusion injury by improving cellular energy metabolism. Liver Transpl. 25, 627–639. 10.1002/lt.25415 30663275

[B27] Mishra P. Chan D. C. (2014). Mitochondrial dynamics and inheritance during cell division, development and disease. Nat. Rev. Mol. Cell. Biol. 15, 634–646. 10.1038/nrm3877 25237825 PMC4250044

[B28] Persad K. L. Lopaschuk G. D. (2022). Energy metabolism on mitochondrial maturation and its effects on cardiomyocyte cell fate. Front. Cell. Dev. Biol. 10, 886393. 10.3389/fcell.2022.886393 35865630 PMC9294643

[B29] Rius-Perez S. Torres-Cuevas I. Millan I. Ortega A. L. Perez S. (2020). PGC-1α, inflammation, and oxidative stress: An integrative view in metabolism. Oxid. Med. Cell. Longev. 2020, 1452696. 10.1155/2020/1452696 32215168 PMC7085407

[B30] Scarpulla R. C. Vega R. B. Kelly D. P. (2012). Transcriptional integration of mitochondrial biogenesis. Trends Endocrinol. Metab. 23, 459–466. 10.1016/j.tem.2012.06.006 22817841 PMC3580164

[B31] Shen H. Wu N. Wang Y. Han X. Zheng Q. Cai X. (2017). JNK inhibitor SP600125 attenuates paraquat-induced acute lung injury: An *in vivo* and *in vitro* study. Inflammation 40, 1319–1330. 10.1007/s10753-017-0575-8 28474156

[B32] Wang X. H. Souders C. L. 2nd Zhao Y. H. Martyniuk C. J. (2018). Paraquat affects mitochondrial bioenergetics, dopamine system expression, and locomotor activity in zebrafish (*Danio rerio*). Chemosphere 191, 106–117. 10.1016/j.chemosphere.2017.10.032 29031050

[B33] Weng C. H. Hu C. C. Lin J. L. Lin-Tan D. T. Hsu C. W. Yen T. H. (2013). Predictors of acute respiratory distress syndrome in patients with paraquat intoxication. PLoS One 8, e82695. 10.1371/journal.pone.0082695 24349340 PMC3859634

[B34] Wilkins H. M. Harris J. L. Carl S. M. E L. Lu J. Eva Selfridge J. (2014). Oxaloacetate activates brain mitochondrial biogenesis, enhances the insulin pathway, reduces inflammation and stimulates neurogenesis. Hum. Mol. Genet. 23, 6528–6541. 10.1093/hmg/ddu371 25027327 PMC4271074

[B35] Wilkins H. M. Koppel S. Carl S. M. Ramanujan S. Weidling I. Michaelis M. L. (2016). Oxaloacetate enhances neuronal cell bioenergetic fluxes and infrastructure. J. Neurochem. 137, 76–87. 10.1111/jnc.13545 26811028 PMC5482267

[B36] Witschi H. Kacew S. Hirai K. I. Cote M. G. (1977). *In vivo* oxidation of reduced nicotinamide-adenine dinucleotide phosphate by paraquat and diquat in rat lung. Chem. Biol. Interact. 19, 143–160. 10.1016/0009-2797(77)90027-8 22404

[B37] Zhang L. Li Q. Liu Z. Wang Y. Zhao M. (2019). The protective effects of bone mesenchymal stem cells on paraquat-induced acute lung injury via the muc5b and ERK/MAPK signaling pathways. Am. J. Transl. Res. 11, 3707–3721. 31312382 PMC6614636

[B38] Zhang Z. D. Yang Y. J. Liu X. W. Qin Z. Li S. H. Li J. Y. (2021). Aspirin eugenol ester ameliorates paraquat-induced oxidative damage through ROS/p38-MAPK-mediated mitochondrial apoptosis pathway. Toxicology 453, 152721. 10.1016/j.tox.2021.152721 33592258

[B39] Zhang Z. Nian Q. Chen G. Cui S. Han Y. Zhang J. (2020). Klotho alleviates lung injury caused by paraquat via suppressing ROS/P38 MAPK-regulated inflammatory responses and apoptosis. Oxid. Med. Cell. Longev. 2020, 1854206. 10.1155/2020/1854206 32509139 PMC7244968

[B40] Zhu J. Wang K. Z. Chu C. T. (2013). After the banquet: Mitochondrial biogenesis, mitophagy, and cell survival. Autophagy 9, 1663–1676. 10.4161/auto.24135 23787782 PMC4028332

[B41] Zyoud S. H. (2018). Investigating global trends in paraquat intoxication research from 1962 to 2015 using bibliometric analysis. Am. J. Ind. Med. 61, 462–470. 10.1002/ajim.22835 29537078

