# Supplementary material for: Oxaloacetate acid ameliorates paraquat-induced acute lung injury by alleviating oxidative stress and mitochondrial dysfunction
Source: Front Pharmacol. 2022 Oct 13;13:1029775. doi: 10.3389/fphar.2022.1029775 (PMC9606601; doi:10.3389/fphar.2022.1029775)
Supplement: Supplementary file 1 [file DataSheet1.docx]

**Supplementary material:**

**
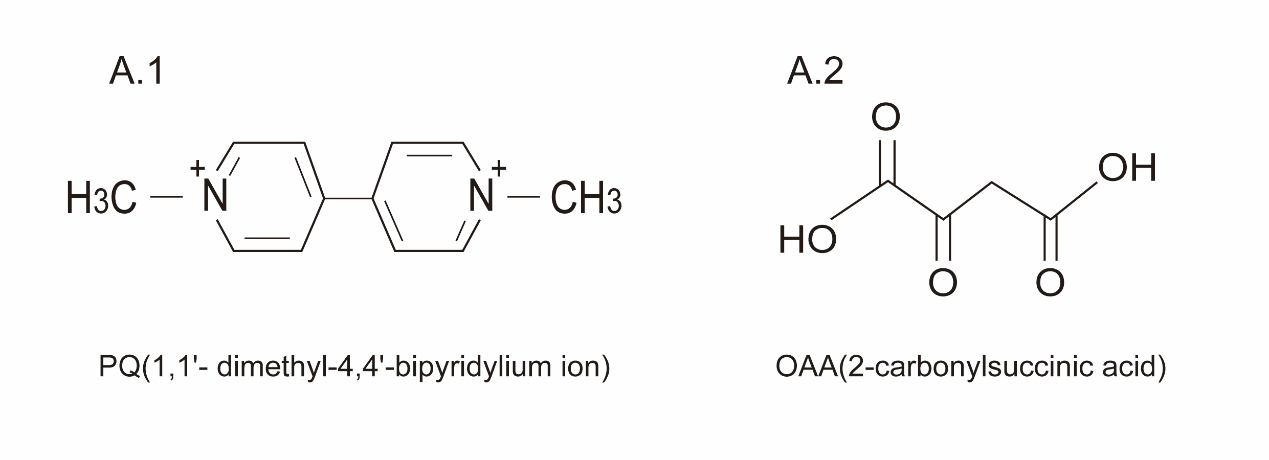
**

**Fig. A** Chemical structures of PQ (1) and OAA (2).

**
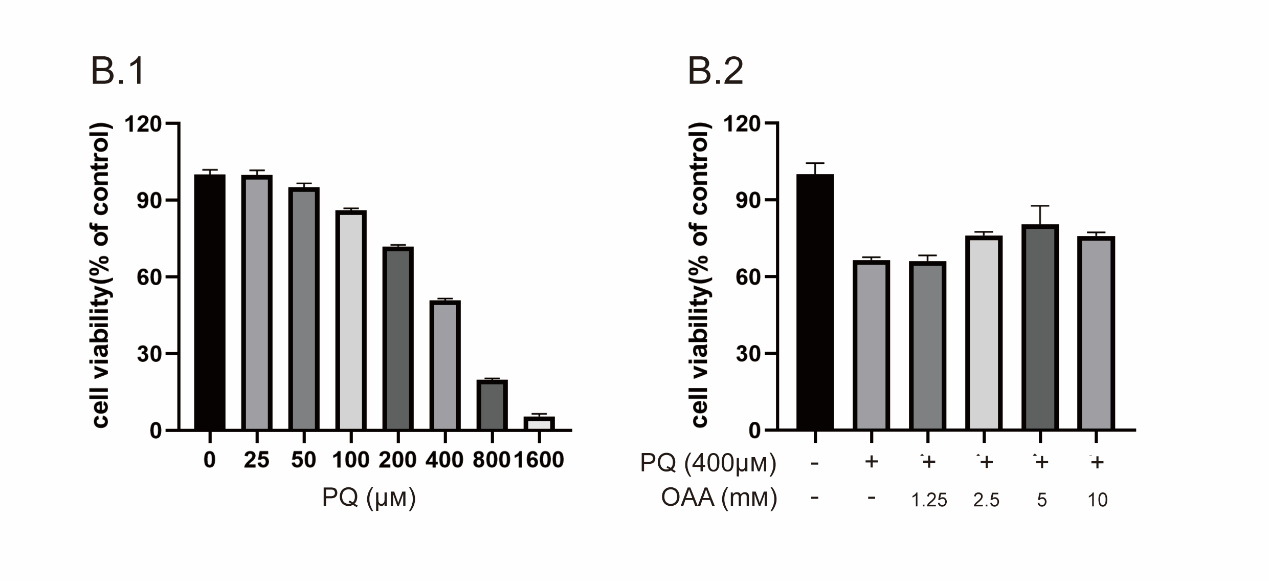
**

**Fig. B** OAA promoted the viability of B2B cells. (1) The cell viability was evaluated after cells were treated with various PQ concentrations. (2) OAA improved cell viability. Data are presented as mean±SD (n=3). ^*^p＜0.05, ^**^p＜0.01, ^***^p＜0.001, ^****^p＜0.0001. PQ, paraquat; OAA, oxaloacetate acid
